# Studies on enhancing operational stability of a reusable laccase-based biosensor probe for detection of ortho-substituted phenolic derivatives

**DOI:** 10.1007/s13205-015-0292-7

**Published:** 2015-03-10

**Authors:** C. Sarika, K. Rekha, B. Narasimha Murthy

**Affiliations:** 1 Department of Biotechnology Engineering, CMR Institute of Technology, Bangalore, 560 037 India; 2 Department of Chemistry, CMR Institute of Technology, Bangalore, 560 037 India

**Keywords:** Amperometric, Biosensor, Co-cross-linking, Laccase

## Abstract

An amperometric principle-based biosensor containing immobilized enzyme laccase from *Trametes versicolor* was developed for detection of ortho-substituted phenolic derivatives. Different immobilization methods for *Trametes versicolor* laccase enzyme on cellophane membrane and the enhancement of operational stability of the immobilized enzyme electrode using various protein-based stabilizing agents were studied. Among tested methods of immobilization, co-cross-linking method with bovine serum albumin was superior to the other methods in terms of sensitivity, limit of detection, response time, and operating and thermal stability. Biosensor response reached steady state within 3 min and exhibited maximum activity at 45 °C and pH 6.8. The sensitivity of the ortho-substituted phenols for the test biosensor developed with co-cross-linking method of immobilization using bovine serum albumin as the protein-based stabilizing agent was in the order: 2-aminophenol > guaiacol(2-methoxyphenol) > catechol(2-hydroxyphenol) > cresol(2-methyl phenol) > 2-chlorophenol. Validation of the newly developed biosensor by comparison with HPLC showed good agreement in the results. A newly developed biosensor was applied for quantification of ortho-substituted phenols in simulated effluent samples.

## Introduction

Phenolic compounds belong to organic pollutants, which are widely distributed in the environment. They may be present in wastewaters and natural environmental waters. Phenols are introduced to the environment in a variety of ways like wastes from paper manufacture, agriculture, petrochemical industry and coal processing or as municipal wastes (Adamski et al. 2010). The phenolic micropollutants generally include chloro-, bromo-, nitro- and alkylphenols (Kim and Kim 2000). Phenols are also breakdown products from natural organic compounds such as humic substances, lignins and tannins. Certain phenols and related aromatic compounds are highly toxic, carcinogenic and allergenic; therefore their determination and removal from the environment are of great importance. Due to health and ecological risks caused by long- and short-term exposure to these phenolic compounds, there is a considerable interest in their measurements in environmental and food samples. In the past decade, a variety of analytical methods were proposed for determination of phenol and its derivatives in natural environmental waters and wastewaters. The most widely used are gas chromatography (Padilla-Sanchez et al. 2011), high-performance liquid chromatography (Alcudia-Leon et al. 2011) and electrochemical methods (Jing et al. 2011). These methods offer proper selectivity and detection limits, but are not suitable for rapid processing of multiple samples and real-time detection. They involve highly trained operators, time-consuming detection processes and complex pre-treatment steps. The instruments are sophisticated and expensive. Further, the methods are unsuitable for field studies and in situ monitoring of samples (Suri et al. 2009).

Recent research activity has focused on the design and construction of biosensors which are capable of improving the efficiency of site monitoring and can be used for the necessary remediation activities. Biosensors use new techniques to build sensors that mix high biological specificity and many possibilities of electronic circuits. These seem to be potential tools to supplement the techniques that are in use, due to particular characteristics as selectivity, low building and storage cost, miniaturizing potential, easy automaticity and simple to build and portable apparatus (Cummings et al. 2001; Chang et al. 2002).

Horseradish peroxidase (HRP) (Korkut et al. 2008; Yin et al. 2009), tyrosinase (Mai Anh et al. 2002; Nadifiyine et al. 2013) and laccase-based electrodes (Portaccio et al. 2006; Shimomura et al. 2011) have been shown to be useful for the selective determination of phenols in environmental matrices as well as total polyphenolic content in food samples (Diaconu et al. 2010; Eremia et al. 2013). In the case of HRP, the limitation is the necessity of the presence of hydrogen peroxide to complete the biocatalytic cycle. The monitoring of the enzyme reaction is accomplished by the electrode reduction of the phenoxy radicals formed, the current being proportional to the concentration of phenolic compounds as long as the H_2_O_2_ concentration is not limiting. Therefore, an excess of H_2_O_2_ should be added to the working solution for the biosensor to be able to respond to the phenolic compounds (Serra et al. 2001). However, it is well known that the presence of a high concentration of H_2_O_2_ causes inhibition of the activity of peroxidase.

A general problem for many tyrosinase biosensors is the lack of the necessary operational and storage stability needed for commercial exploitation and is currently a major obstacle to be solved in the biosensor area. The instability of tyrosinase biosensors in pure standard solutions is mainly because quinones suffer from high instability in water and formation of intermediate radicals in both enzymatic and electrochemical reactions. Radicals can react and polymerize to polyaromatics, which can inactivate the biocatalyst and foul the electrode (Kotte et al. 1995). Laccase is one of the best candidates for use in analytical systems for the determination of phenolic compounds (Gianfreda et al. 1999) mainly due to its great stability, a wide range of detectable phenolic compounds and insusceptibility to product inhibition. Laccases (benzenediol:oxygen oxidoreductase, E.C.1.10.3.2) are copper-containing oxidoreductases produced by higher plants and microorganisms, mainly fungi. Laccases reduce oxygen directly to water in a four-electron transfer step without intermediate formation of soluble hydrogen peroxide at the expense of one-electron oxidation of a variety of substrates, e.g. phenolic compounds (Haghighi et al. 2003).

Enzyme stabilization and its storage are important criteria in biosensor development (D’souza 2001). Immobilization is a commonly used method for enzyme stabilization and storage, and it can be reused many times. The procedures used for immobilization of enzymes on different substrates and their advantages and disadvantages have been widely discussed in many reviews of nanoscale materials (Pingarron et al. 2008; Li et al. 2014) and conducting polymers (Gerard et al. 2002; Ahuja et al. 2007). A review has been devoted to immobilization of laccase (Lac) and tyrosinase (Tyr) on different substrates (Duran et al. 2002). However, immobilization is costly and requires an inert matrix. For practical biosensor application to become a reality, inexpensive methods of immobilizations should be obtained. An optimal immobilization procedure should ensure activity and stability of the protein and, at the same time, provide good accessibility of substrate molecules to the active site of the enzyme. Retention of biological activity of the enzyme molecule is paramount and depends on the stabilization of the biological structure of the enzyme. In most cases, the mechanism of stabilization is a little understood phenomenon. As far as we are aware, this is the first extensive study on enhancement of operational stability of a *Trametes versicolor* laccase-based biosensor for the detection of ortho-substituted phenolic compounds using various methods of enzyme immobilization with different protein-based stabilizing agents (PBSAs).

## Materials and methods

### Reagents

Laccase (from *Trametes versicolor*) having specific activity 10 IU mg^−1^ was procured from Sigma (USA); 2-aminophenol, catechol (2-hydroxy phenol), guaiacol (2-methoxy phenol), cresol (2-methyl phenol) and 2-chlorophenol were from SRL Chem., India. Appropriate amount of various phenols were dissolved in double-distilled water to give the desired concentration. Bovine serum albumin (BSA), lysozyme, gelatin and cellophane membrane were procured from Himedia and glutaraldehyde from SD Fine Chem., India. All reagents were of analytical grade and used as received. Double-distilled water was used throughout the experiments.

### Apparatus

A Clark’s electrode with an amperometric detection system was used for detection of substituted phenols. An amperometric principle-based detector system developed in our laboratory was used to amplify and monitor the signals obtained from the enzyme electrode. Clark type of dissolved oxygen electrode was purchased from M/S Century Instruments, Chandigarh, India. The Clark-type electrode consists of a gold (Au) cathode and a reference Ag/AgCl electrode covered with saturated KCl electrolyte enclosed within a Teflon membrane. A polarizing potential of −650 mV was applied to the gold working electrode.

### Enzyme immobilization procedure

Various methods of immobilization such as (1) physical adsorption, (2) cross-linking, (3) entrapment in gelatin and (4) hybrid method, i.e. entrapment along with cross-linking were done on cellophane membrane. All enzyme membranes were kept at room temperature for 1 h for drying.

#### Physical adsorption

Physical adsorption of the laccase was done directly on cellophane membrane. 2 IU enzyme was taken on the cellophane membrane and kept at room temperature until all the water was allowed to evaporate.

#### Cross-linking with glutaraldehyde

2 IU of enzyme was cross-linked using glutaraldehyde (2 %) on cellophane membrane.

#### Co-cross-linking with various PBSAs

2 IU of enzyme was cross-linked with glutaraldehyde (5 %) on cellophane membrane using 4 mg of PBSAs, i.e. BSA, lysozyme and gelatin.

#### Entrapment in gelatin

Entrapment in gelatin was done by mixing gelatin (4 mg) with 2 IU enzyme on the surface of the cellophane membrane.

#### Hybrid method

2 IU enzyme was mixed with 2 mg gelatin, 2 mg BSA and 5 % glutaraldehyde and spread on a cellophane membrane.

### Construction of biosensor and measurement of response

**Fig. 1:**
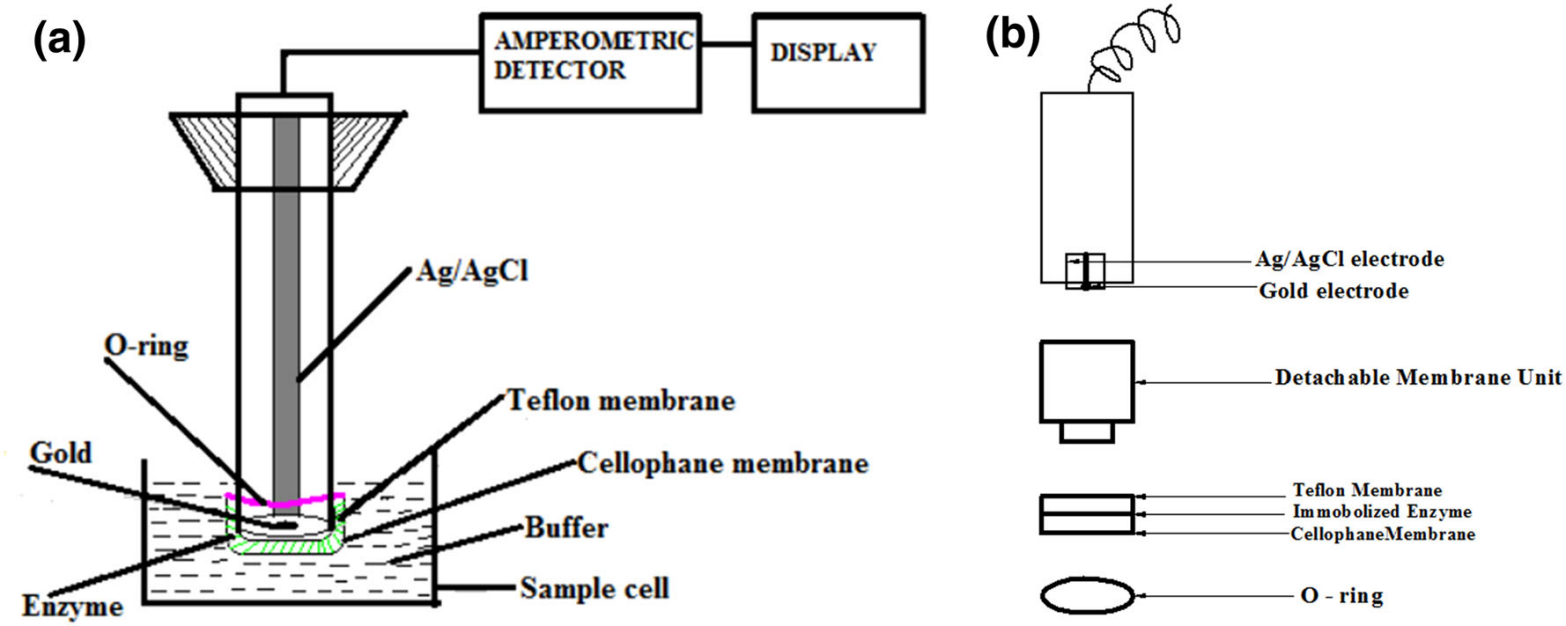
**a** Biosensor configuration. **b** Detachable membrane unit

After drying for 1 h, the enzyme membrane was washed with 0.1 M phosphate buffer (pH 6.8) to remove excess glutaraldehyde. This was attached to the surface of the probe using an inner Teflon membrane by rolling the “O” ring over the end of the probe. This was immersed in a sample cell containing 5 ml of 0.1 M phosphate buffer of pH 6.8. A schematic diagram of the biosensor and its detachable membrane unit is shown in Fig. 1a, b.

The sample cell was continuously saturated with oxygen using a portable air pump. The probe was connected to the amperometric detector system developed in our laboratory. 50 µl of standard solutions of different phenols of varying concentrations was injected into the buffer solution using a micropipette. When the substrate was introduced into the sample cell, enzyme reaction proceeded, resulting in depletion of oxygen in the vicinity of the enzyme membrane, thereby yielding an electrochemical signal of decreasing current. This response was converted into voltage, amplified and monitored and was directly proportional to the concentration of the analyte. The time taken to reach a steady state was 3 min. The system provides a two-point calibration for quantification of various substituted phenols.

### Optimization of parameters for the biosensor performance

#### Optimum pH for biosensor performance

The effect of pH on sensor response was studied by incubating the immobilized enzyme membrane for 30 min in 0.1 M buffer of different pH values in the range 3–8 and the response for catechol (50 µM) was measured at that pH. Acetate buffer was used for acidic range and phosphate buffer for neutral and basic range.

#### Optimum temperature for biosensor performance

The optimum temperature for biosensor performance was determined by incubating the enzyme electrode for the co-cross-linking method with BSA in 0.1 M phosphate buffer, pH 6.8, at temperatures ranging from 27 to 70 °C for catechol (50 µM).

#### Optimum concentration of enzyme

The effect of enzyme loading on biosensor performance was studied taking different enzyme concentrations ranging from 0.5 to 10 IU.

### Operational stability of immobilized enzyme membrane

The operational stability studies of immobilized enzyme electrode were carried out at 28 ± 2 °C. The enzyme membranes were stored at room temperature by keeping them immersed in 0.1 M phosphate buffer of pH 6.8, the activity was checked daily by injecting 50 µM catechol and the response recorded as drift in voltage.

### Mechanism of stabilization of laccase

For this experiment, a 2 IU/ml of free laccase solution was incubated in a shaking water bath (60 rpm) at 60 °C. 20 μl enzyme aliquots were withdrawn from this sample before incubating in the water bath. 20 μl aliquots were also drawn at different time intervals while incubating in a water bath. The enzyme activity was measured. The same experiment was repeated using PBSAs such as BSA, gelatin and lysozyme. These proteins were added to the enzyme solution in such a way that the concentration of the added protein corresponded to 4 mg/ml. This was incubated at room temperature for 1 h to allow protein–protein interaction. 20 μl aliquots was drawn before keeping it in water bath. The sample was kept in a water bath adjusted to 60 °C (60 rpm). 20 μl aliquots were drawn at various time intervals and the enzyme activity was measured.

To study the mechanism of stabilization of laccase by BSA, experiments were carried out in the presence of sodium bromide which disrupts hydrophobic interactions. 1 M NaBr was prepared in 0.1 M phosphate buffer, pH 6.8. This was used to make up the enzyme solution (2 IU/ml). BSA (4 mg/ml) was added to this solution and it was incubated at room temperature for 1 h. This was kept in a shaking water bath at 60 °C. 20 μl aliquots were drawn before keeping it in the water bath and also at various time intervals while incubating in the water bath.

### Studies on immobilized enzyme kinetics

Kinetic parameters for the immobilized laccase catalysed reaction were calculated and compared to arrive at the optimal method of enzyme immobilization.

### Calibration plots and analytical characteristics

Various analytical features such as linearity range, limit of detection, correlation coefficient (*R* ^2^) and sensitivity were studied for different methods of enzyme immobilization.

### Studies on reproducibility

The reproducibility of the biosensor was studied by using the fabricated biosensor for estimating a known concentration of catechol (40–100 µM) in ten replicates each. The observations were statistically analysed for standard deviation (SD) and coefficient of variation (CV).

### Sample application

Validation of the newly developed biosensor was carried out by comparing the results obtained from the test biosensor with that of HPLC. A Shimadzu High Performance Liquid Chromatograph equipped with an LC 20AD model pump and an injector was used in the present study and the column effluents were monitored at 280 nm. Peak areas were determined using LC solution software. The flow rate was 1 ml/min. A 250 × 4.6 mm Luna 5u C18 column was used. Methanol/water mixture was used as the mobile phase.

The simulated effluents were prepared by mixing various ortho-substituted phenolic compounds. The newly developed biosensor was applied for the analysis of ortho-substituted phenols in simulated effluent samples.

## Results and discussion

### Fabrication of detector system

The current generated from the enzyme electrode depends on the analyte concentration, area of electrode and also on the protocol used to develop the biosensing membrane. The current was converted to voltage and amplified using signal conditioning circuit. The resulting voltage is given by *V*_1_ = 10 K × I. In the next stage, the resulting voltage *V*_1_ is further amplified to the voltage range *V*_2_ = (*R*_f_/*R*_2_) *V*_1_, where *R*_f_ = 47 K and *R*_2_ = 100 Ω. *V*_2_ = 470 *V*_1_. The output of the signal conditioning circuit was measured through a multimeter.

### Optimization of parameters for the biosensor performance

#### Optimum pH for biosensor performance

The response to catechol when the immobilized enzyme membrane was incubated in buffers of varying pH values for 30 min was measured. For the enzyme membranes immobilized using various PBSAs, the optimum pH was found to be between 6.5 and 7 (Table 1).

**Table 1:** Optimum pH for immobilized enzyme electrodes

Immobilization method	Optimum pH
Cross-linking	6.5
Co-cross-linking with	
Bovine serum albumin	6.8
Lysozyme	6.8
Gelatin	7
Entrapment	7
Hybrid	6.8

#### Optimum temperature for biosensor performance

**Fig. 2:**
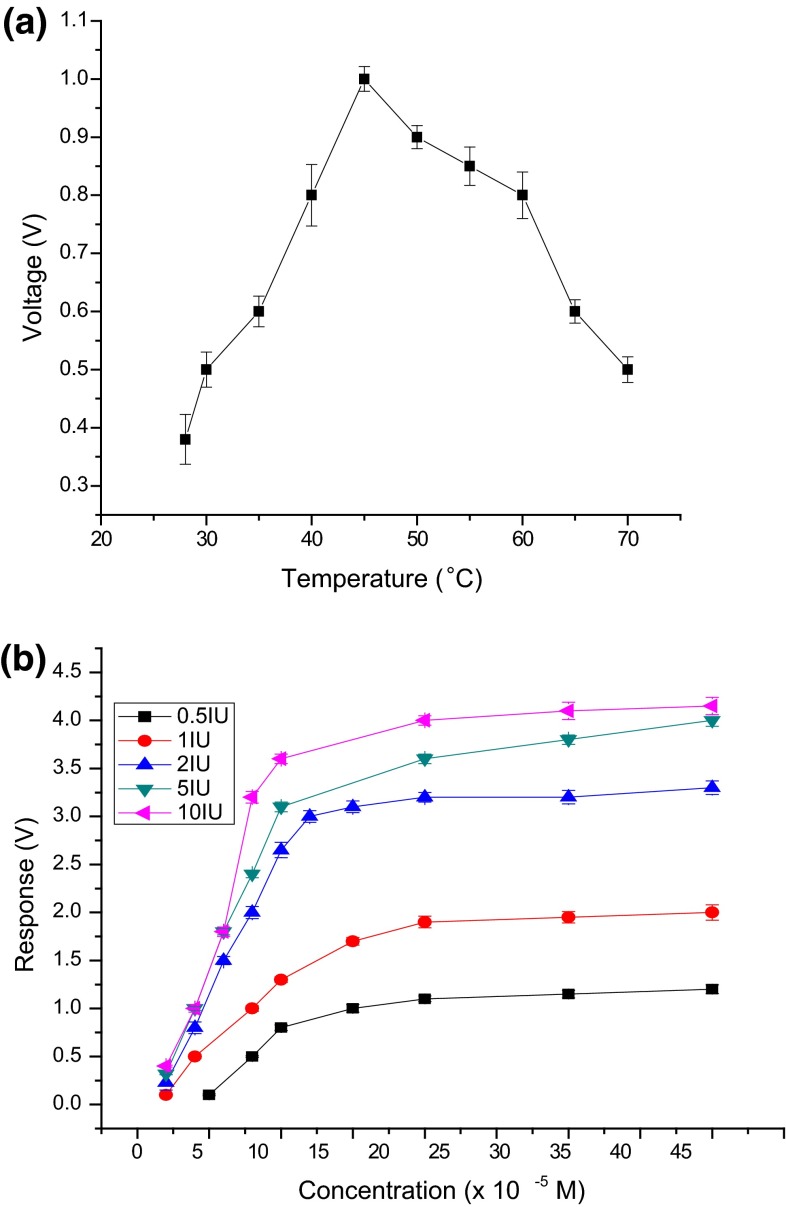
**a** Optimum temperature for biosensor performance. **b** Studies on enzyme loading

For the co-cross-linking method with BSA as PBSA, the response to catechol increased up to 45 °C and later decreased. Therefore, the optimum temperature for biosensor performance was 45 °C (Fig. 2a). The results of the study with *Trametes versicolor* species is almost similar as mentioned in the literature stating that laccase activity is maximum at temperatures between 30 and 50 for enzymes obtained from sources such as *Trametes hirsuta*, *Sclerotium rolfsii* and *Pleurotus ostreatus* (Duran et al. 2002).

#### Optimum concentration of enzyme

The effect of enzyme loading on biosensor performance and optimum concentration of laccase was studied by taking different enzyme concentrations ranging from 0.5 to 10 IU. Catechol concentrations ranging from 1 × 10^−5^ to 40 × 10^−5^M were used. When enzyme concentration increased from 0.5 to 10 IU, response in volts increased linearly with concentration of catechol. Better sensitivity was observed with 2 IU laccase (Fig. 2b). Therefore, 2 IU was selected for further studies.

### Operational stability of immobilized enzyme membrane

**Fig. 3:**
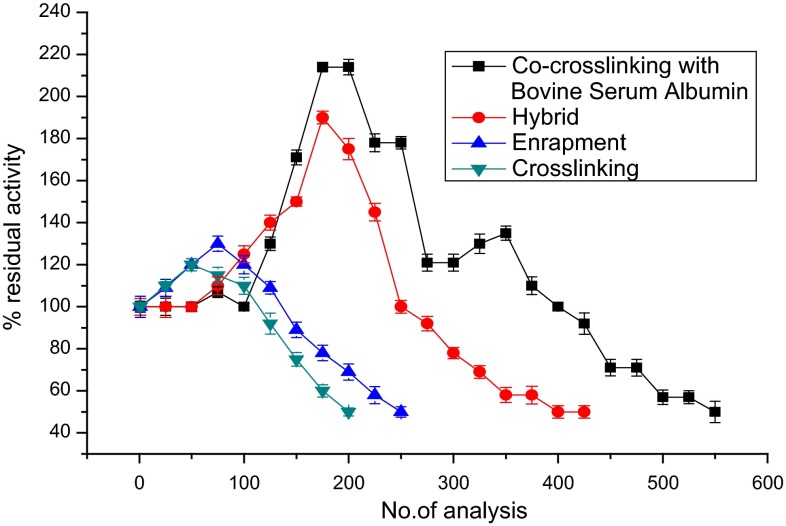
Operational stability of laccase-based biosensor employing various methods of immobilization

The operational stability studies of different immobilized enzyme membranes were carried out at room temperature (28 ± 2 °C). Figure 3 demonstrates the operational stability of the enzyme electrode for various immobilization methods. To quantify the operational stability of various enzyme immobilization methods, repeated measurements with 50 µM catechol were carried out. As shown in the figure, as the number of analyses increased, the activity of immobilized laccase (in terms of biosensor response) increased initially probably owing to the decreased diffusional barriers for the analyte which resulted from changes of the enzyme’s 3D structure inside the immobilization matrix upon rehydration (Khan and Wernet 1997). Further usage of immobilized enzyme for repeated analysis of catechol leads to sharp decrease in activity. However, enzyme immobilized using the co-cross-linking method with BSA as PBSA showed a high stable activity up to 200 analyses and thereafter declined to reach 50 % activity around 550 analyses. Thus, co-cross-linking using BSA as PBSA was found to be the best method for immobilization of laccase.

**Fig. 4:**
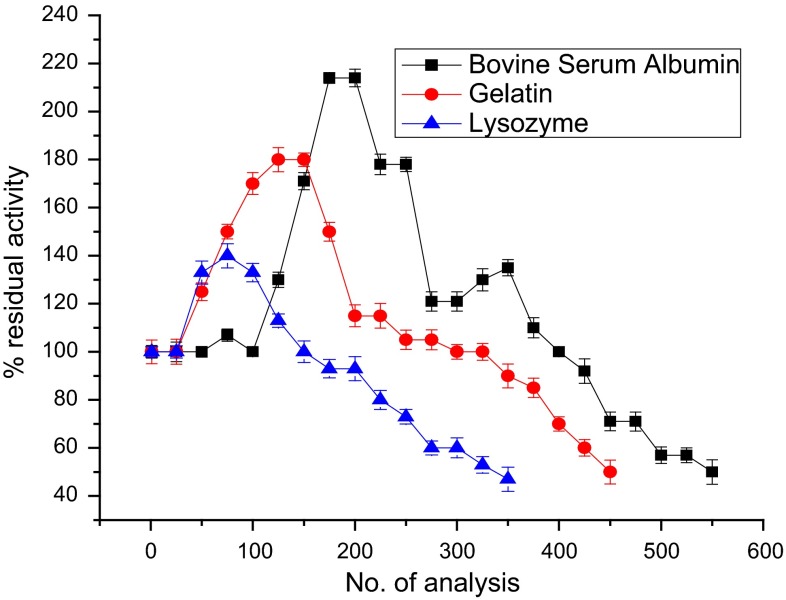
Operational stability of laccase-based biosensor constructed using the co-cross-linking method of immobilization using different PBSAs

The order of operational stability for the different methods of immobilization is as follows: co-cross-linking > hybrid > entrapment > cross-linking (Fig. 3; Table 2). Among the PBSAs tested, BSA was found to be the best as evident in (Fig. 4; Table 3). The order of operational stability for the different PBSAs is as follows: BSA > gelatin > lysozyme.

**Table 2:** Comparison of % activity retained after repeated analysis of catechol for the different immobilizations

Immobilization method	% Activity retained after no. of analyses for 50 µM catechol						No. of analyses possible with 50 % activity retention (half-life)
	100	200	300	400	500	550	
Co-cross-linking with bovine serum albumin	100	214	121	100	57	50	550
Entrapment	120	69	–	–	–	–	250
Cross-linking	110	50	–	–	–	–	200
Hybrid	113	165	80	65	–	–	450

**Table 3:** % Activity retained after repeated analysis for laccase electrode immobilized using different protein-based stabilizing agents

Protein-based stabilizing agents	% Activity after no. of analyses for 50 µM catechol						No. of analyses possible with 50 % activity retention (half life)
	100	200	300	400	500	550	
Bovine serum albumin	100	214	121	100	57	50	550
Gelatin	170	115	100	70			450
Lysozyme	133	93	60				350

The storage stability of enzyme membrane co-cross-linked with BSA was investigated by measuring 50 µM catechol, three to four analyses per day, and the detachable membrane units were stored at 4 °C by immersing in 0.1 M phosphate buffer of pH 6.8 when not in use. The enzyme membrane retained 70 % of its initial activity over a period of 7 months, which is comparable to covalently immobilized sensors (Freire et al. 2001).

### Mechanism of stabilization of laccase

**Fig. 5:**
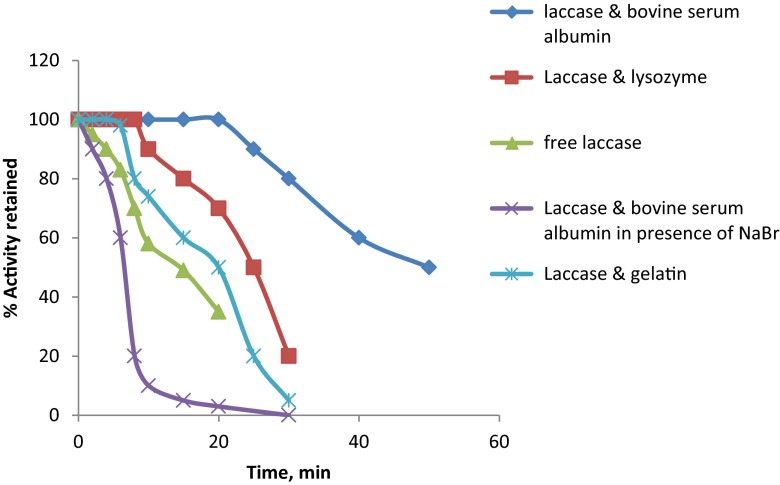
Temperature-induced enzyme inactivation of soluble enzyme in the presence of various PBSAs and NaBr

Studies conducted on various immobilization methods showed that the co-cross-linking method of immobilization using BSA yielded best results in terms of operational stability and activity. Further studies conducted on the mechanism of stabilization of laccase by various PBSAs showed that BSA stabilized the enzyme by means of hydrophobic interactions, as BSA could not stabilize the enzyme in the presence of NaBr. Soluble enzyme in the presence of BSA could retain 100 % activity for 20 min when incubated at 60 °C, whereas the free enzyme could retain only 35 % activity after 20 min incubation at 60 °C (Fig. 5).

### Thermal stability of immobilized laccase

**Fig. 6:**
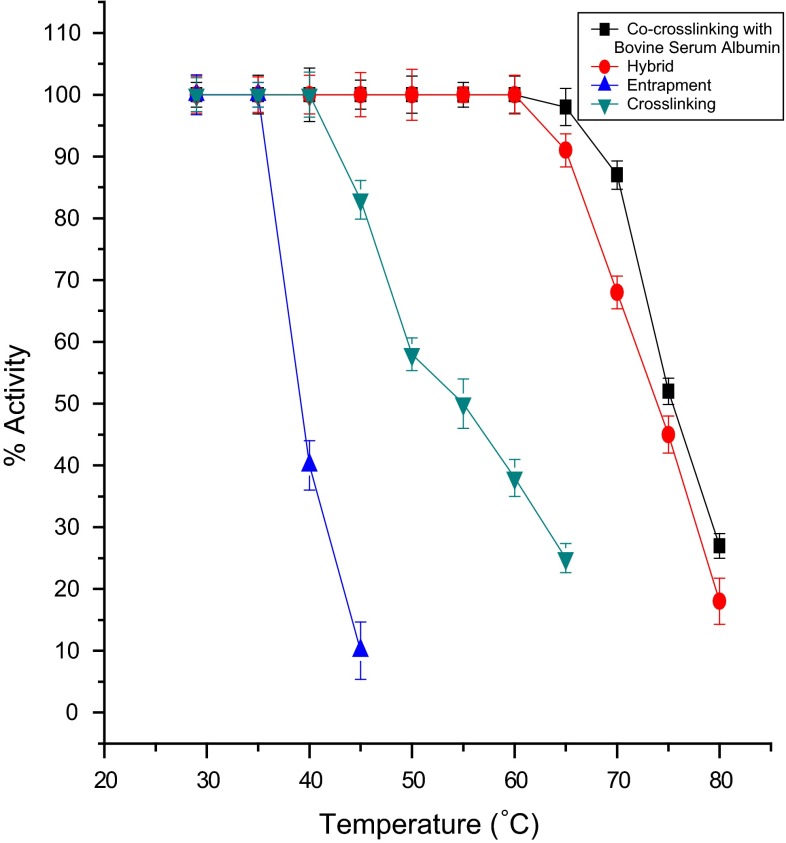
Thermal inactivation of laccase-based biosensor using various methods of immobilization

**Fig. 7:**
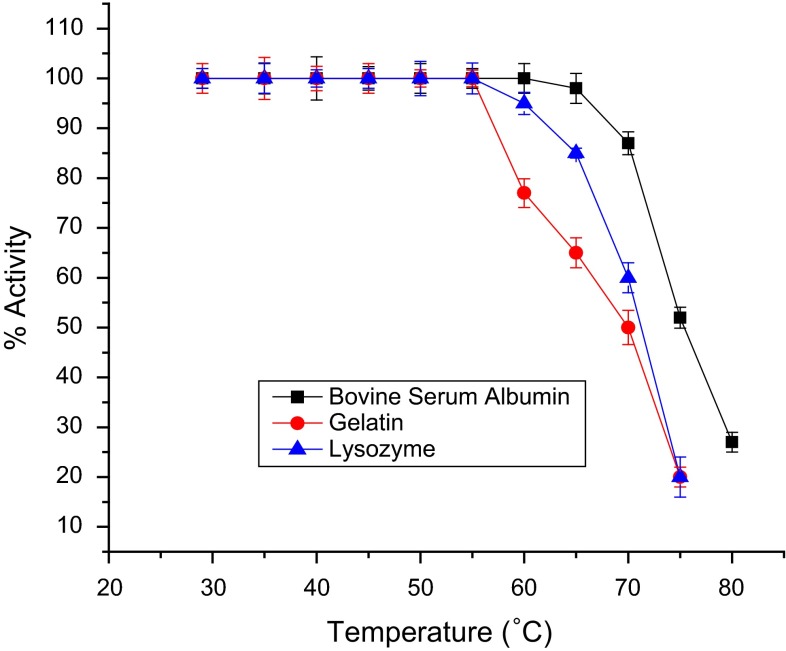
Thermal inactivation of laccase-based biosensor using the co-cross-linking method of immobilization with different PBSAs

For thermal stability studies on immobilized enzyme membranes, freshly immobilized enzyme membranes were kept at 28 ± 2 °C overnight in 0.1 M buffer solution of pH 6.8. The initial activity ‘a’ of the enzyme membrane for 50 µM of catechol was measured at 28 ± 2 °C. The electrode was then immersed for 15 min in 5 ml buffer at the desired temperature using a constant temperature bath. The enzyme membrane was immediately cooled to room temperature in an ice bath for 2 min. Then the residual activity ‘b’ of the immobilized enzyme was observed at room temperature by injecting 50 µM of catechol. The % residual activity was calculated as [*b*/*a*] × l00. Thus, the residual activity was calculated at different temperatures in the range of 40–80 °C in steps of 5 °C. Figures 6 and 7 demonstrate the thermal deactivation behaviour of laccase-based biosensor for various methods of immobilization and also for co-cross-linking method of immobilization using various PBSAs (gelatin, BSA and lysozyme) at pH 6.8. Co-cross-linking with BSA could retain 100 % activity, whereas lysozyme and gelatin retain 95 and 77 %, respectively, at 60 °C. This showed that the co-cross-linking method of immobilization using BSA as PBSA exhibited the highest thermal stability.

### Immobilized enzyme kinetics

Immobilized enzyme kinetics was studied using catechol as a model substrate. Apparent Michaelis–Menten constants (*K*_m app._) and the maximum rate of reaction (*V*_max_) were calculated from the corresponding Lineweaver–Burk plots as shown in (Table 4).

**Table 4:** Kinetic parameters for the laccase-catalysed reaction using enzyme electrode employing various methods of immobilization with catechol as substrate

Immobilization method	*V*_max_ (V)	*K*_m app._ (×10^−5^ M)	*V*_max._ (V)/*K*_m app._ (×10^−5^ M)
Co-cross-linking with			
Bovine serum albumin	2.8	5.75	0.4870
Lysozyme	3.05	11.87	0.2570
Gelatin	3.5	16.59	0.2109
Cross-linking	3.25	30.57	0.1063
Entrapment	3.1	17.89	0.1733
Hybrid	2.95	15.95	0.1850

*K*_m app._ values were lower for co-cross-linking with BSA when compared with other immobilization techniques. *K*_m app._ is an indicator of the affinity that an enzyme has for a given substrate and, hence, the stability of the enzyme–substrate complex. The kinetics of laccase-catalysed reactions is first affected by the affinity between enzyme and the substrate. An estimation of this influence can be done by amperometric measurements in terms of the *V*_max_/*K*_m app._ ratio. Co-cross-linking with BSA and glutaraldehyde displays a greater affinity towards catechol when compared with other methods of immobilization.

**Fig. 8:**
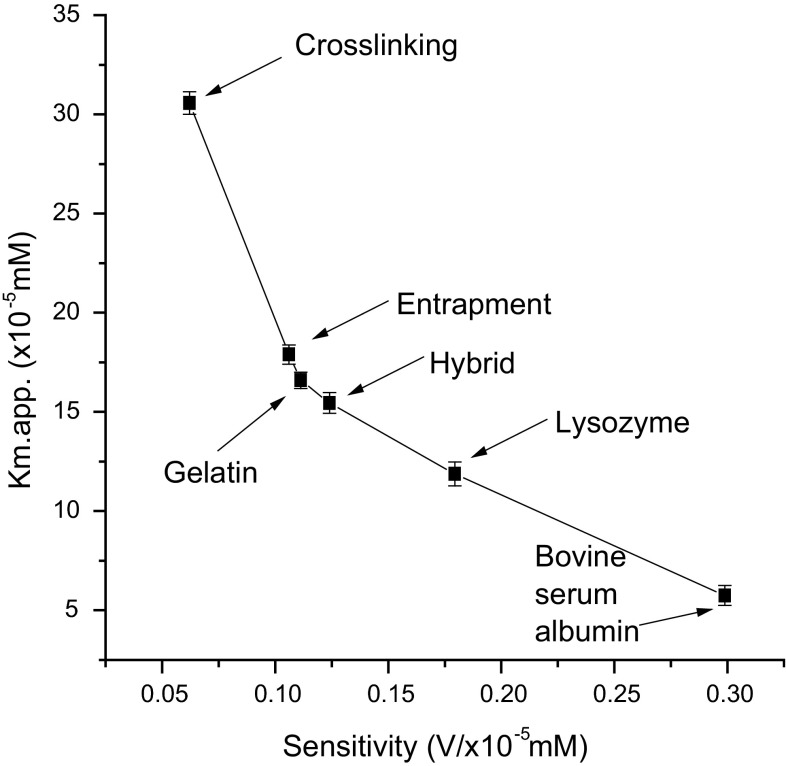
K_m app._ values as a function of sensitivity

When *K*_m app._ values are reported as a function of sensitivities, the results reported in (Fig. 8) emerge. The data in Fig. 8 clearly show how small values of sensitivity correspond to small affinity values, i.e. to high values of *K*_m app._ and vice versa. It is therefore evident that, to design laccase-based biosensors for the determination of phenolic compounds, one must adapt the immobilization methods on the basis of the interest in having high sensitivity.

### Calibration plots and analytical characteristics

Table 5 summarizes the characteristics of the calibration plots obtained for the different ortho-substituted phenolic compounds tested with laccase electrodes using different methods of immobilization under the optimized working conditions of temperature and pH.

**Table 5:** Calibration data and analytical characteristics for the different ortho-substituted phenolic compounds using laccase biosensor employing various immobilization methods

Method of immobilization	Enzyme may have leached out before the start of analysis					
	LOD (×10^−5^ M)	Response time min.	Recovery time min.	Linearity range (×10^−5^ M)	*R* ^2^	Slope
Physical adsorption						
Cross-linking						
Guaiacol	3	4	1	4–40	0.9910	0.0961
2-aminophenol	1	4	1	2–30	0.9891	0.191
Catechol	4	5	1.15	4–50	0.993	0.0621
2-methylphenol	13.7	5	1.20	10–60	0.9902	0.0521
2-Chlorophenol	16.2	5	1.15	25–100	0.9962	0.0397
Co-cross-linking with bovine serum albumin						
Guaiacol	0.5	3	0.5	0.6–10	0.9928	0.3047
2-aminophenol	0.3	3	0.5	0.5–8	0.9950	0.3527
Catechol	0.7	3	0.5	1–10	0.9934	0.2988
2-methylphenol	1	4	1	2–16	0.9893	0.2092
2-Chlorophenol	6	4	1	6–18	0.9806	0.1523
Co-cross-linking with lysozyme						
Guaiacol	1	4	1	2–10	0.9941	0.2991
2-aminophenol	0.8	4	1	1–10	0.9908	0.3092
Catechol	2	5	1.10	2–20	0.9923	0.1795
2-methylphenol	4	6	1.30	4–40	0.9958	0.1336
2-Chlorophenol	10	6	1.30	20–80	0.9891	0.0935
Co-cross-linking with gelatin						
Guaiacol	1.2	5	1.30	2–20	0.9922	0.195
2-Aminophenol	0.9	5	1.30	2–10	0.9991	0.2253
Catechol	3	7	1.40	3–30	0.9902	0.1113
2-methylphenol	5	7	2	6–50	0.9962	0.1081
2-Chlorophenol	12	8	2	20–100	0.9904	0.0465
Entrapment						
Guaiacol	2	5	2	2–30	0.9961	0.1643
2-Aminophenol	1.2	5	2	2–20	0.9922	0.2099
Catechol	4	7	2	4–35	0.9896	0.1061
2-Methylphenol	5	7	2	6–50	0.9907	0.0695
2-Chlorophenol	10	8	2	20–100	0.9962	0.051
Hybrid						
Guaiacol	1.5	4	1	2–20	0.9890	0.2811
2-aminophenol	0.5	4	1	0.6–10	0.9915	0.3013
Catechol	3	6	1.30	3–30	0.9920	0.1241
2-methylphenol	4.5	7	1.50	6–20	0.9982	0.0923
2-Chlorophenol	6.8	7	1.50	8–40	0.9981	0.0791

**Fig. 9:**
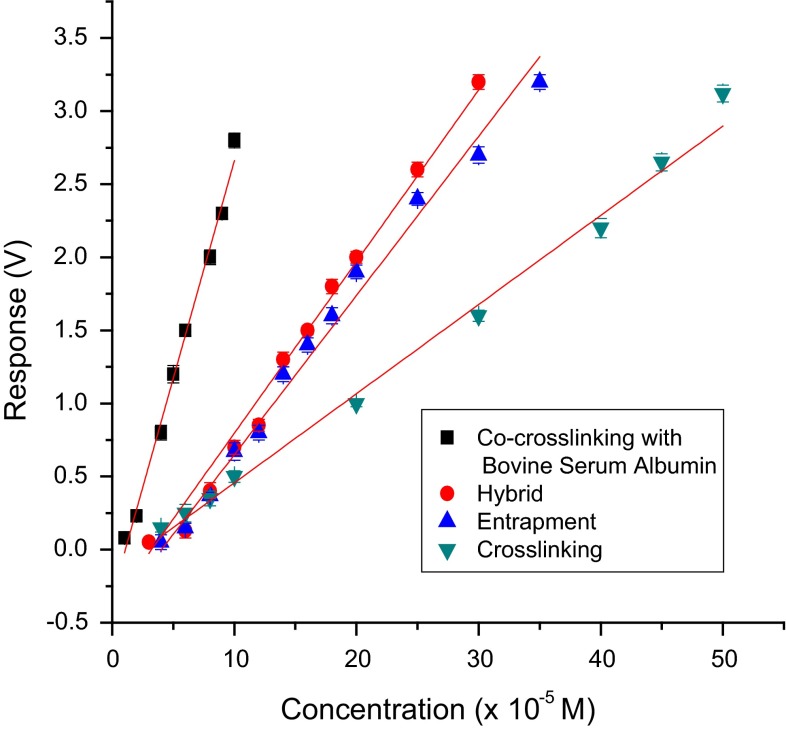
Calibration graph for catechol using biosensor employing various immobilization methods

**Fig. 10:**
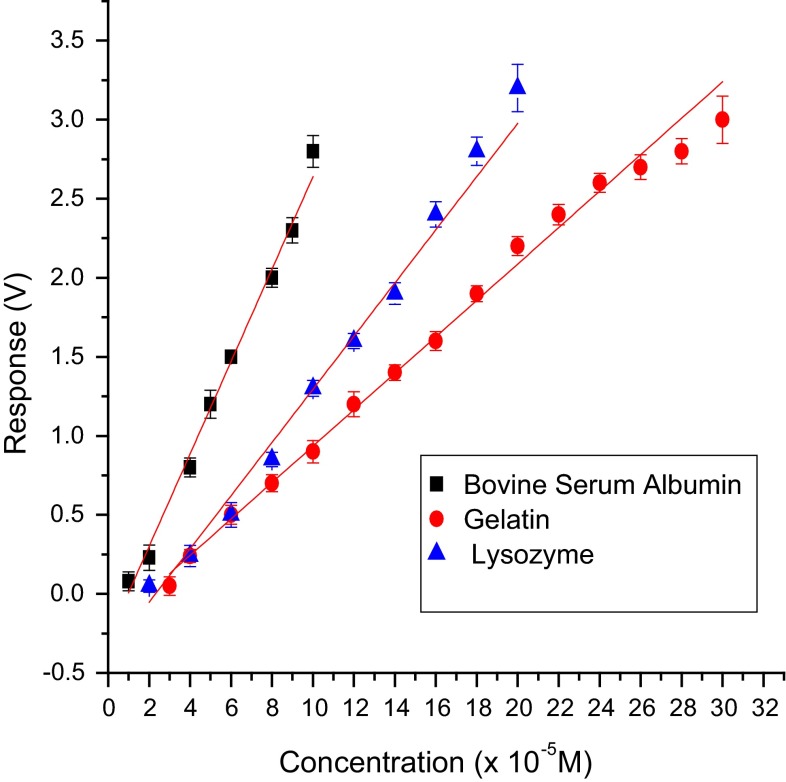
Calibration graph for catechol with test biosensor using laccase cross-linked with various PBSAs

Sensitivity of the test biosensor developed with various methods of immobilization using different PBSAs with catechol as model substrate was found to be in the order: co-cross-linking with BSA > co-cross-linking with lysozyme > co-cross-linking with gelatin > hybrid > entrapment in gelatin > cross-linking with glutaraldehyde (Figs. 9, 10). Therefore, the co-cross-linking method with BSA as PBSA was found to be the best in terms of sensitivity, limit of detection (LOD) and response time. For all immobilization methods except cross-linking, biosensor response and sensitivity are inversely related to the thickness of the membrane which is evident from Table 6. A sharp decrease in the activity of immobilized laccase for the cross-linking method is probably due to inactivation of the enzyme with excess glutaraldehyde.

**Table 6:** Characteristics of enzyme membranes

Immobilization method	Membrane thickness (µ)
Cross-linking	116.25
Co-cross-linking with	
Bovine serum albumin	133.25
Lysozyme	145.00
Gelatin	168.78
Entrapment	175.00
Hybrid	185.75

**Fig. 11:**
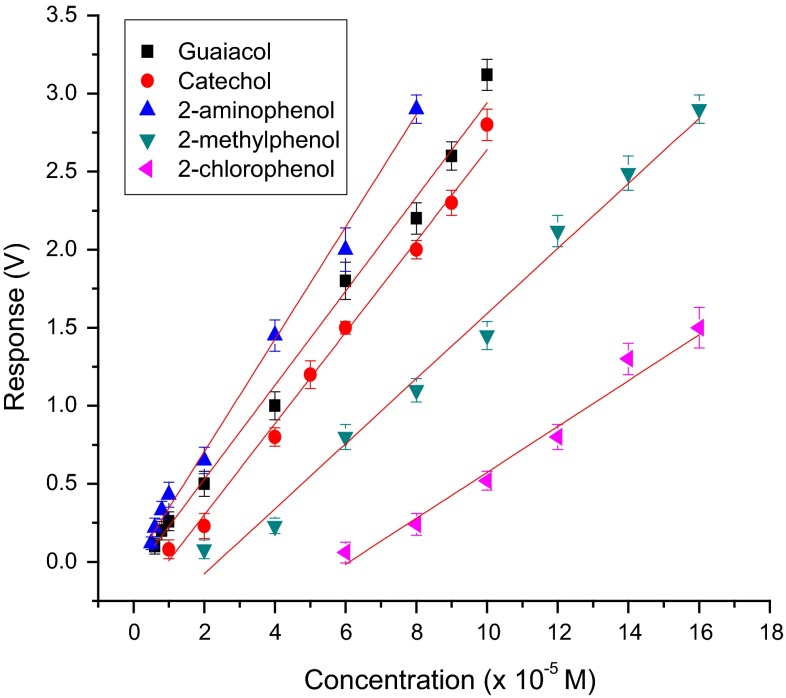
Calibration graph for ortho-substituted phenolic compounds with the co-cross-linking method of immobilization using BSA as PBSA

The sensitivity using this sensor for ortho-substituted phenolic compounds was in the order: 2-aminophenol > guaiacol (2-methoxyphenol) > catechol (2-hydroxyphenol) > cresol (2-methyl phenol) > 2-chlorophenol (Fig. 11). It was also noticed that laccase from *Trametes versicolor* did not respond to O-nitrophenol. The electron-donating power of the substituents for various phenolic compounds tested is in the order: –NH2 > –OCH3 > –OH > –Cl > –NO2. As the nitro group is a strongly electron-withdrawing substituent, the methoxy, methyl and hydroxyl substituents are strongly electron donating and the chloro group is a weakly electron-donating substituent, these observations also suggest that the phenolic ring has to be electron rich for oxidation by *Trametes versicolor* laccase.

In Table 7, the characteristics of the proposed biosensor containing laccase enzyme immobilized using the co-cross-linking method with BSA as PBSA are compared with other laccase-based catechol and catecholamine biosensors found in the literature. This suggests that the performance characteristics and operating stability of the proposed biosensor are comparable or better to other previously reported laccase-based electrodes.

**Table 7:** A comparison of properties of various laccase-based catechol and catecholamine biosensors

Nature of electrode	Substrate	LOD (µM)	Linear range (µM)	Operating stability (no. of cycles)	References
ITO/PANI/Lac	Catechol	NR	0.4–15	175	Kushwah et al. (2011)
SPE/MWCNT/Si/Lac	Dopamine (catecholamine)	0.42	1.3–85.5	20	Li et al. (2012)
CLECs of laccase entrapped in PVP gel	Catechol	NR	0.05–0.5	30	Jegan Roy et al. (2005)
Covalent immobilization of laccase on Pt electrode	Dopamine (catecholamine)	0.2	0.5–40	600	Quan and Shin (2004)
Laccase co-cross-linked with BSA and glutaraldehyde	Catechol	7	10–100	550	Present work

*NR* not reported

### Reproducibility

To check the reproducibility and accuracy of the newly developed biosensor, a known concentration of catechol was taken (40–100 µM) in ten replicates each and the fabricated biosensor was used for its estimation.

The observations were statistically analysed for standard deviation (SD) and coefficient of variation (CV) given in Table 8. The results showed that the determinations were consistent in terms of reproducibility, accuracy and reusability for all methods of immobilizations.

**Table 8:** Statistical analysis of biosensor response to varying concentrations of catechol for the various types of immobilizations

Type of immobilization	Concentration of catechol (µM)	Standard deviation (SD)	Coefficient of variation (CV)
Cross-linking	40	0.0055	0.0375
	60	0.0084	0.0337
	80	0.0071	0.02020
	100	0.0114	0.0226
Co-cross-linking with (bovine serum albumin)	40	0.011	0.01365
	60	0.0195	0.01294
	80	0.0228	0.0114
	100	0.0182	0.00649
Co-cross-linking with (lysozyme)	40	0.0084	0.0351
	60	0.0071	0.0141
	80	0.0045	0.00524
	100	0.013	0.009937
Co-cross-linking with (gelatin)	40	0.0084	0.0351
	60	0.00957	0.019
	80	0.005	0.00714
	100	0.023	0.02569
Entrapment	40	0.0019	0.0374
	60	0.01587	0.0988
	80	0.0148	0.0398
	100	0.0245	0.0371
Hybrid	40	0.0038	0.0246
	60	0.0045	0.0338
	80	0.013	0.0141
	100	0.023	0.019

### Sample application

Validation of the test biosensor was successfully performed by comparing the results with conventional HPLC. The proposed biosensor containing laccase enzyme immobilized using co-cross-linking method with BSA as PBSA was applied for the analysis of phenolic compounds in simulated effluents. 50 µl of simulated effluents were added to the reaction cell after equilibration had occurred and then the change in voltage was measured. The signals obtained from the effluent samples were found to be very similar with that of the reference phenolic compound solutions having the same concentration. Good correlation was observed between results obtained with the test biosensor and those with HPLC (Table 9).

**Table 9:** Simulated effluent sample analysis using test biosensor

Sample	Different phenol contents	Phenolic compound content (theoretical) (µg/ml)	Phenolic compound content (experimental) (µg/ml)	
			Biosensor	HPLC
Simulated effluent 1	Guaiacol 0.022 µg Catechol 0.48 µg Cresol 0.02 µg	0.52	0.51 ± 0.033 (1.9)	0.52 (0)
Simulated effluent 2	2-Aminophenol 0.30 µg 2-Chlorophenol 0.24 µg	0.54	0.50 ± 0.049 (7.4)	0.53 (1.9)
Simulated effluent 3	Catechol 0.22 µg 2-Aminophenol 0.04 µg	0.26	0.27 ± 0.027 (3.8)	0.26 (0)
Simulated effluent 4	Guaiacol 0.022 µg Catechol 0.22 µg 2-Aminophenol 0.073 µg	0.315	0.32 ± 0.038 (1.5)	0.31 (1.5)

Percentage error of instrumental methods in comparison with theoretical values is represented in parenthesis. Results are expressed as ±SD, *n* = 5

The error was found to be <4 % for all samples, with biosensor except for sample no 2, and this may be due to the decreased sensitivity of 2-chlorophenol to laccase biosensor as evident from Fig. 11. Therefore, the system could be easily applied for the screening of ortho-substituted phenolic compounds in industrial wastewaters.

## Conclusions

Sensitive, rapid and precise determination of phenols and its derivatives is important in environmental control and protection. As the biosensor technique starts moving from proof-of-concept stage to field testing under realistic processes or waste-monitoring conditions, the need for the availability of stable biological materials will become important (D’souza 2001). Disposable sensors can be active for a few minutes, whereas reusable sensors require several days to several months of stability. Reusable probes are more economical from the operational point of view. When immobilized enzymes are used for this purpose, the activity loss due to denaturation and deactivation of enzyme diminishes the life of the sensor. Therefore techniques to enhance the storage and operational stability of the enzyme electrode are important in the application of electrochemical biosensors (Gouda et al. 2002).

An important limitation in the application of immobilized enzymes in various biotechnological processes is their low thermal and operational stability. Thermal stability of enzymes is important for use in immobilized enzyme-based biosensors (Sarath Babu et al. 2004). Thermal denaturation of enzymes is mainly due to the destabilization of ionic and hydrophobic interactions and breakage of hydrogen bonds, van der Waals forces and ionic interactions which lead to a conformational change in the tertiary structure of the enzyme and thus render it inactive. Stabilization of the desired enzyme can be achieved by using certain proteins, which may be catalytically active or inactive (Gouda et al. 2002).

Inclusion of additives is as effective in elevating enzyme stability as protein engineering, and does not require the sheer time and effort (with associated costs) as the protein engineering regime. Though reports are available on stabilization of enzymes for amperometric biosensor applications using positively charged soluble polymer (diethyl aminoethyl dextran) and neutral carbohydrate (lacitol) (Gibson et al. 1992), still there is scope for further improvement through better understanding of the mechanism of inactivation. The main causes for thermal inactivation of enzymes are thiol group oxidation, denaturation and finally aggregation (Ye and Combes 1989). Denaturation and aggregation of enzymes can be minimized by using additives like salts, polyols, polyelectrolytes and immobilization through protecting tertiary structure. Further by incorporating appropriate protein-based stabilizing agents (PBSA) during immobilization, the thermal stability of the immobilized enzymes can be improved significantly (Gouda et al. 2003). Most of the recent literature on biosensors revolve around the use of disposable screen-printed electrodes (Eremia et al. 2013; Li et al. 2012). Proteins can be liable to damage and denaturation when in solution for extended periods, irrespective of whether the sensor is laid down by screen printing, biodotting or ink jetting. The drying process, i.e. extraction of moisture from the enzyme solution on the sensor surface, is probably the major step in the process which will lead to the inactivation of a majority of proteins.

In this paper, the influence of different enzyme immobilization techniques on cellophane membrane on the performances of laccase-based Clark-type electrodes has been evaluated. The kinetic analytical properties and thermal behaviour of the resulting biosensors were tested with different ortho-substituted substrates. One of the suitable enzyme immobilization methods is cross-linking using glutaraldehyde. Glutaraldehyde being a strong bifunctional reagent modifies the enzyme drastically, leading to conformational changes and loss of activity. This deleterious effect can be minimized using inert proteins such as BSA, gelatin and thrombin. These proteins avoid excessive intermolecular cross-linkages within the enzyme and enhance intermolecular linkages between enzyme and inert proteins (Broun et al. 1973). In our study, we have found that co-cross-linking with BSA could retain 100 % activity whereas lysozyme and gelatin retain 95 and 77 %, respectively, at 60 °C for 15 min. Soluble enzyme in the presence of BSA could retain 100 % activity for 20 min when incubated at 60 °C, whereas the free enzyme could retain only 35 % activity after 20 min incubation at 60 °C. Enzyme stabilization using the co-cross-linking method with BSA as PBSA shows a high stable activity up to 200 analyses and thereafter declined to reach 50 % activity around 550 analyses. In this work, we have found that the incorporation of BSA during the process of immobilization contributes to long-term stability of laccase-based biosensor. Results indicate that co-cross-linking method with BSA was superior to other methods of immobilization in terms of sensitivity, LOD, response time and operating stability. The reason for the better performance of co-cross-linking method of immobilization with BSA may be because BSA stabilizes the enzyme by hydrophobic interactions. Hydrophobic interactions are considered as the single most important factor in stabilization of enzyme structure. Therefore, strengthening of these interactions should impart structural rigidity to the enzyme molecules and thus make them more resistant to unfolding.

This indicates that the system can be used in applications at higher temperatures, involving protein stabilization studies and also in biosensors for online monitoring. This method can also be used in the preparation of heat-sterilizable probes for food and fermentation analysis. Our results are hopefully a significant step towards understanding the action of inert proteins in enhancing the stability of immobilized enzymes.

Improvements in both operational and storage stability indicate that the methodology is relatively generic in nature and can be adopted for many application areas. Work is in progress to predict the mechanism of stabilization with the aim of being able to predict the type of stabilizer needed for the specific enzyme. Data accumulated from such experiments will help us to understand more about how proteins denature at molecular level and ultimately enable us to stabilize enzymes in a more predictable fashion. Moreover, the detachable membrane unit used by us for these studies has enabled a convenient method to follow the activity of the immobilized enzyme over a long duration using a number of enzyme membranes, but a single dissolved oxygen probe. This has made relatively large number of analyses possible, in a convenient and economical way. The biosensor technique has been useful to track the operational stability of the immobilized enzymes used in this study. With a combination of expertise in field enzyme stabilization and advances in methods used for predicting enzyme structural changes during the degradation process, it will become increasingly easy to predict how to stabilize an enzyme for specific industrial application.

Sensitivity of the ortho-substituted phenolic compounds using co-cross-linking method with BSA was in the order: 2-aminophenol > guaiacol(2-methoxyphenol) > catechol(2-hydroxyphenol) > cresol(2-methyl phenol) > 2-chlorophenol. Results indicated that laccase immobilized using co-cross-linking method with BSA as PBSA could be successfully used for the detection of ortho-substituted phenolic compounds in industrial effluent samples. The use of bi-enzyme systems employing laccase and tyrosinase may be attempted, as these two enzymes display different substrate selectivity and mechanisms, and thus the bi-enzyme-based biosensors allow the detection of different phenolic compounds in environmental samples.

## References

[CR1] Adamski J, Nowak P, Kochana J (2010). Simple sensor for determination of phenol and its derivatives in water based on enzyme tyrosinase. Electrochim Acta.

[CR2] Ahuja T, Mir IA, Kumar D, Rajesh (2007). Biomolecular immobilization on conducting polymers for biosensing applications. Biomaterials.

[CR3] Alcudia-Leon MC, Lucena R, Cardenas W, Valcarcel M (2011). Determination of phenols in waters by stir membrane liquid - liquid - liquid microextraction coupled to liquid chromatography with ultraviolet detection. J Chromatogr A.

[CR4] Broun G, Thomas D, Gellf G, Domurado D, Berjonneau AM, Guillon C (1973). New methods for binding enzyme molecules into a water-insoluble matrix: properties after insolubilization. Biotechnol Bioeng.

[CR5] Chang SC, Rawson K, Mc Neil CJ (2002). Disposable tyrosinase- peroxidase bi-enzyme sensor for amperometric detection of phenols. Biosens Bioelectron.

[CR6] Cummings EA, Linquette-Mailley S, Mailley P, Cosnier S, Eggins BR, McAdams ET (2001). A Comparison of amperometric screen-printed, carbon electrodes and their application to the analysis of phenolic compounds present in beers. Talanta.

[CR7] D’souza SF (2001). Immobilization and stabilization of biomaterials for biosensor applications. Appl Biochem Biotechnol.

[CR8] De Quan, Shin W (2004). Amperometric detection of catechol and catecholamines by immobilized laccase from denilite. Electroanalysis.

[CR9] Diaconu M, Litescu SC, Radu GL (2010). Laccase-MWCNT-chitosan biosensor-A new tool for total polyphenolic content evaluation from in vitro cultivated plants. Sens Actuators B: Chem.

[CR10] Duran N, Rosa MA, D’Annibale A, Gianfreda L (2002). Applications of laccases and tyrosinases (phenoloxidases) immobilized on different supports: a review. Enzym Microb Technol.

[CR11] Eremia SAV, Vasilescu I, Litescu SC, Radu GL (2013). Disposable biosensor based on platinum nanoparticles-reduced graphene oxide-laccase biocomposite for the determination of total polyphenolic content. Talanta.

[CR12] Freire RS, Duran N, Kubota LT (2001). Effects of fungal laccase immobilization procedures for the development of a biosensor for phenol compounds. Talanta.

[CR13] Gerard M, Chaubey A, Malhorta BD (2002). Application of conducting polymers to biosensors. Biosens Bioelectron.

[CR14] Gianfreda L, Xu F, Bollag JM (1999). Laccases: a useful group of oxidoreductive enzymes. Biorem J.

[CR15] Gibson TD, Higgins IJ, Woodward JR (1992). Stabilization of analytical enzymes using a novel polymer-carbohydrate system and the production of a stabilized, single reagent for alcohol analysis. Analyst.

[CR16] Gouda MD, Kumar MA, Thakur MS, Karanth NG (2002). Enhancement of operational stability of an enzyme biosensor for glucose and sucrose using protein based stabilising agents. Biosens Bioelectron.

[CR17] Gouda MD, Singh SA, Appu Rao AG, Thakur MS, Karanth NG (2003). Thermal inactivation of glucose oxidase: mechanism and stabilisation using additives. J Biol Chem.

[CR18] Haghighi B, Gorton L, Ruzgas T, Jönsson LJ (2003). Characterization of graphite electrodes modified with laccase from *Trametes versicolor* and their use for bioelectrochemical monitoring of phenolic compounds in flow injection analysis. Anal Chim Acta.

[CR19] Jegan Roy J, Emilia Abraham T, Abhijith KS, Sujith Kumar PV, Thakur MS (2005). Biosensor for the determination of phenols based on cross-linked enzyme crystals (CLEC) of laccase. Biosens Bioelectron.

[CR20] Jing T, Xia H, Niu J, Zhou Y, Dai Q, Hao Q, Zhou Y, Mei S (2011). Determination of trace 2,4-dinitrophenol in surface water samples based on hydrophilic molecularly imprinted polymers/nickel fiber electrode. Biosens Bioelectron.

[CR21] Khan GF, Wernet W (1997). Design of enzyme electrodes for extended use and storage life. Anal Chem.

[CR22] Kim K-R, Kim H (2000). Gas chromatographic profiling and screening for phenols as isobutoxycarbonyl derivatives in aqueous samples. J Chromatogr A.

[CR23] Korkut S, Keskinler B, Erhan E (2008). An amperometric biosensor based on multiwalled carbon nanotube-poly (pyrrole)-horseradish peroxidase nanobiocomposite film for determination of phenol derivatives. Talanta.

[CR24] Kotte H, Gruendig B, Vorlop K-D, Strehlitz B, Stottmeister U (1995). Methylphenazonium modified enzyme sensor based on polymer thick films for subnanomolar detection of phenols. Anal Chem.

[CR25] Kushwah BS, Upadhyaya SC, Shukla S, Sikarwar AS, Sengar RMS, Bhadauria S (2011). Performance of nanopolyaniline-fungal enzyme based biosensor for water pollution. Adv Mat Lett.

[CR26] Li Y, Zhang L, Li M, Pan Z, Li D (2012). A disposable biosensor based on immobilization of laccase with silica spheres on the MWCNTs-doped screen-printed electrode. Chem Cent J.

[CR27] Li D, Luo L, Pang Z, Ding L, Wang Q, Ke H, Huang F, Wei Q (2014). Novel phenolic biosensor based on a magnetic polydopamine-laccase-nickel nanoparticle loaded carbon nanofiber composite. ACS Appl Mater Interfaces.

[CR28] Mai Anh T, Dzyadevych SV, Soldatkin AP, Duc Chien N, Jaffrezic-Renault N, Chovelon J-M (2002). Development of tyrosinase biosensor based on pH-sensitive field-effect transistors for phenols determination in water solutions. Talanta.

[CR29] Nadifiyine S, Haddam M, Mandli J, Chadel S, Blanchard CC, Marty JL, Amine A (2013). Amperometric biosensor based on tyrosinase immobilized on to a carbon black paste electrode for phenol determination in olive oil. Anal Lett.

[CR30] Padilla-Sanchez JA, Plaza-Bolanos P, Romero-Gonzalez R, Barco-Bonilla N, Martinez-Vidal JL, Garrido-Frenich A (2011). Simultaneous analysis of chlorophenols, alkylphenols, nitrophenols and cresols in wastewater effluents, using solid phase extraction and further determination by gas chromatography-tandem mass spectrometry. Talanta.

[CR31] Pingarron JM, Yanez-Sadeno P, Gonzales-Cortes A (2008). Gold nanoparticle-based electrochemical biosensors. Electrochim Acta.

[CR32] Portaccio M, Di Martino S, Maiuri P, Durante D, De Luca P, Lepore M, Bencivenga U, Rossi S, De Maio A, Mita DG (2006). Biosensors for phenolic compounds: the catechol as a substrate model. J Mol Catal B Enzym.

[CR33] Sarath Babu VR, Kumar MA, Karanth NG, Thakur MS (2004). Stabilisation of immobilised glucose oxidase against thermal inactivation by silanisation for biosensor applications. Biosens Bioelectron.

[CR34] Serra B, Benito B, Agui L, Reviejo AJ, Pingarron JM (2001). Graphite-teflon-peroxidase composite electro-chemical biosensors. A tool for the wide detection of phenolic compounds. Electroanalysis.

[CR35] Shimomura T, Itoh T, Sumiya T, Hanaoka T-A, Mizukami F, Ono M (2011). Amperometric detection of phenolic compounds with enzyme immobilized in mesoporous silica prepared by electrophoretic deposition. Sens Actuators: B Chem.

[CR36] Suri CR, Boro R, Nangia Y, Gandhi S, Sharma P, Wangoo N, Rajesh K, Shekhawat GS (2009). Immunoanalytical techniques for analyzing pesticides in the environment. Trends Anal Chem.

[CR37] Ye WN, Combes D (1989). The relationship between the glucose oxidase subunit structure and its thermostability. Biochim Biophys Acta.

[CR38] Yin H, Ai S, Shi W, Zhu L (2009). A novel hydrogen peroxide biosensor based on horseradish peroxidase immobilized on gold nanoparticles-silk fibroin modified glassy carbon electrode and direct electrochemistry of horseradish peroxidase. Sens Actuators B: Chem.

